# *Streptococcus suis* 2 Transcriptional Regulator TstS Stimulates Cytokine Production and Bacteremia to Promote Streptococcal Toxic Shock-Like Syndrome

**DOI:** 10.3389/fmicb.2018.01309

**Published:** 2018-06-19

**Authors:** Zhongmin Xu, Bo Chen, Qiang Zhang, Liang Liu, Anding Zhang, Yujie Yang, Kaisong Huang, Shuxian Yan, Junping Yu, Xiaomei Sun, Meilin Jin

**Affiliations:** ^1^Unit of Animal Infectious Diseases, National Key Laboratory of Agricultural Microbiology, College of Veterinary Medicine, Huazhong Agricultural University, Wuhan, China; ^2^Key Laboratory of Preventive Veterinary Medicine in Hubei Province, The Cooperative Innovation Center for Sustainable Pig Production, Wuhan, China; ^3^Key Laboratory of Development of Veterinary Diagnostic Products, Ministry of Agriculture, Wuhan, China

**Keywords:** *Streptococcus suis*, transcriptional regulator, STSLS, bacteremia, excessive inflammation

## Abstract

Two large-scale outbreaks of streptococcal toxic shock-like syndrome (STSLS) have revealed *Streptococcus suis* 2 to be a severe and evolving human pathogen. We investigated the mechanism by which *S. suis* 2 causes STSLS. The transcript abundance of the transcriptional regulator gene *tstS* was found to be upregulated during experimental infection. Compared with the wild-type 05ZY strain, a *tstS* deletion mutant (Δ*tstS*) elicited reduced cytokine secretion in macrophages. In a murine infection model, *tstS* deletion resulted in decreased virulence and bacterial load, and affected cytokine production. Moreover, TstS expression in the P1/7 strain of *S. suis* led to the induction of STSLS in the infected mice. This is noteworthy because, although it is virulent, the P1/7 strain does not normally induce STSLS. Through a microarray-based comparative transcriptomics analysis, we found that TstS regulates multiple metabolism-related genes and several virulence-related genes associated with immune evasion.

## Introduction

*Streptococcus suis* is a serious threat to the swine industry and human health worldwide (Gottschalk et al., 2013; Feng et al., 2014; Segura et al., 2014). *S. suis* has 29 serotypes, among which *S. suis* type 2 (SS2) is recognized as the most infectious and pathogenic serotype for animals and humans (Wisselink et al., 2000; Hill et al., 2005; Tien le et al., 2013; Feng et al., 2014; Kerdsin et al., 2016). SS2 recently caused two large-scale outbreaks of streptococcal toxic shock-like syndrome (STSLS) in China with over 200 human cases and nearly 20% fatality (Yu et al., 2006). Patients exhibited a high fever, shock, a clear erythematous blanching rash, and multiple organ failure (Tang et al., 2006; Yu et al., 2006). Massive cytokine production is the main feature of STSLS. Compared with the European strain P1/7, the highly virulent Chinese SS2 strains (98HAH33 and 05ZYH33) stimulate a greater production of proinflammatory cytokines such as interleukin-6 (IL-6), interleukin-12p70 (IL-12p70), and tumor necrosis factor α (TNF-α) in humans, mice, and pigs (Dominguez-Punaro et al., 2007; Bi et al., 2014). These cytokines can serve as indicators of STSLS (Dominguez-Punaro et al., 2007; Zhao et al., 2011).

Unlike the European P1/7 strain, the highly virulent Chinese SS2 strains contain an 89K pathogenicity island (PAI), which is a genomic island likely acquired through horizontal gene transfer that contributes to virulence and may be associated with STSLS (Chen et al., 2007). Further analysis of the 89K PAI identified two two-component signal-transduction systems and six stand-alone transcriptional regulators, of which the two two-component signal transduction systems have been proven to be essential for the full virulence of the highly virulent Chinese SS2 strains (Okwumabua and Chinnapapakkagari, 2005; Li et al., 2008). It is known that numerous stand-alone transcriptional regulators such as Mga, CcpA, and CodY also influence the virulence of streptococci (Hondorp and McIver, 2007; Li et al., 2007; Willenborg et al., 2014; Feng et al., 2016). In the present work, all six stand-alone transcriptional regulators of the 89K PAI were studied. Among them, SSU05_0930 was obviously upregulated *in vivo* and its knock-out mutant elicited a reduced secretion of cytokines by macrophages. Thus, we hypothesized that the transcriptional regulator TstS, which is encoded by SSU05_0930 and has the full name “Toxic Shock-like related Transcription regulators of *S. suis*,” may promote STSLS. We also studied the effects of introducing *tstS* into the P1/7 strain as we conjectured that other strains may cause some pathologies of STSLS after acquiring *tstS* through horizontal gene transfer.

## Materials and Methods

### Bacterial Strains, Plasmids, and Growth Conditions

The *S. suis* strains used in this study are listed in **Table 1**. Strain 05ZY, which has the 89K PAI and causes STSLS, was chosen as the wild-type strain (Li et al., 2010). Strain P1/7 is the European strain of highly virulent *S. suis* and has the characteristics of lacking the 89K PAI and not causing STSLS.

**Table 1:** Bacterial strains used in this study.

*S. suis* strains	Characteristics	Reference
05ZY	Highly virulent strain isolated from the brain of a diseased piglet collected during the 2005 Sichuan outbreak in China; produces STSLS to pigs or mice; has an intact 89K island	Laboratory collection
05ZY-pSET2	The 05ZY strain with the empty plasmid pSET2	This study
Δ*tstS*	05ZY derivative with the *tstS* knockout	This study
Δ*tstS*-pSET2	The ΔtstS strain with the empty plasmid pSET2	This study
CΔ*tstS*	The ΔtstS strain with the plasmid pSET2 carrying *tstS*	This study
P1/7	European reference strain of highly virulent *S. suis* serotype 2 isolated from a field case of meningitis	ATCC (BAA-853)
P1/7-pSET2	The P1/7 strain with an empty plasmid pSET2	This study
P1/7-*tstS*	The P1/7 strain with the plasmid pSET2 carrying the *tstS* gene	This study

A temperature-sensitive *S. suis Escherichia coli* shuttle vector that carries the spectinomycin resistance gene (*spe*), pSET4s, was used to construct the *tstS* knockout mutant (Takamatsu et al., 2001). An *S. suis E. coli* shuttle vector, pSET2, which also carries *spe*, was used to construct the complementary and TstS expression strains.

All the *S. suis* strains were grown in tryptic soy broth medium or plated on tryptic soy agar (Difco, Detroit, MI, United States) containing 5% (vol/vol) newborn bovine serum at 37°C (Yang et al., 2015a). *E. coli* strains were cultured in Luria–Bertani broth or on Luria–Bertani agar at 37°C. When required, 50 μg/ml spectinomycin was added for *E. coli* and 100 μg/ml spectinomycin was added for *S. suis* (Takamatsu et al., 2001).

### Cell Culture and Infection

Cells were grown in Dulbecco’s modified Eagle’s medium (DMEM) supplemented with 10% fetal bovine serum in a 5% CO_2_ atmosphere at 37°C. Primary mouse macrophages were prepared as previously described (Zhang et al., 2017). The BALB/c mice were injected intraperitoneally with 4% thioglycolate. Four days later, peritoneal exudate cells were harvested and identified by microscopy with non-specific esterase staining (Sodhi et al., 2005). When >90% of the exudate cells were identified as macrophages, the cells were plated at a density of 10^6^ cells per well in 12-well plates for *in vitro* pathogenicity tests (Sodhi et al., 2005). Cells were infected with 5 × 10^6^ colony forming units (CFUs) per well of each strain in the logarithmic phase of growth for 6 h, and then collected for RNA extraction.

### RNA Isolation and qPCR Analysis

*In vivo* bacteria were separated from mouse or piglet blood as previously described for RNA isolation. Piglet blood from previous studies was stored in liquid nitrogen. One hour before inoculation, four pigs with a good health status (age range 4–5 weeks) were given 2 ml of 1% acetic acid (pH 2.9) intranasally to enhance the sensitivity of the SS2 challenge. The pigs were inoculated intranasally with 1 ml of 2 × 10^8^ CFUs of strain 05ZY. All the pigs that presented typical symptoms were sacrificed and dissected (Li et al., 2010). Blood was harvested and immediately frozen in liquid nitrogen.

Specific pathogen-free female BALB/c mice were intraperitoneally infected with 05ZY. At 6 h post-inoculation (h.p.i.), the mice that presented typical symptoms were sacrificed and dissected. Blood was harvested and immediately frozen in liquid nitrogen.

To investigate the transcript abundance of *tstS* in human blood, 1 × 10^8^ CFUs of 05ZY was inoculated into 2 ml human blood and incubated for 2 h at 37°C.

Prior to RNA isolation, the blood samples (500 μl) were combined with 500 μl of an ice-cold solution composed of 0.4 M sucrose and 0.01% sodium dodecyl sulfate (SDS). The mixture was gently centrifuged for 10 min at 300 rpm twice to remove large cell debris. The suspension was centrifuged for 15 min at 5,000 rpm to harvest the bacteria (Camejo et al., 2009).

*In vitro* bacteria were grown at 37°C in tryptic soy broth medium with shaking until they reached the mid-log phase (optical density at 600 nm = 0.7) and were then harvested by centrifugation for 5 min at 4,000 rpm.

Total bacterial RNA was extracted as previously described (Zhang et al., 2015). Relative quantitative PCR (qPCR) was performed using a SYBR^®^ Green PCR kit (Roche, Basel, Switzerland) on a ViiA7 thermal cycler (Applied Biosystems, Foster City, CA, United States) with three biological replicates. 16S rDNA was used as the reference gene. Data were analyzed using the QuantStudio^TM^ software (Applied Biosystems) (Zhang et al., 2015).

### Construction of Knockout Mutants, Complementation Strains, TstS Expression Strains, and Control Strains

#### pSET4s-ΔtstS

Two ∼600-bp flanking fragments of the *tstS* open reading frame (ORF) were amplified from the 05ZY genome using the primer pairs *tstS*L-1/*tstS*L-2 and *tstS*R-1/*tstS*R-2 (Supplementary Table S1). The two fragments were digested with the EcoRI, BamHI, SalI, and HindIII enzymes, and then cloned into pSET4s to form the knockout plasmid pSET4s-Δ*tstS*.

#### pSET2-TstS

The DNA fragment containing the *tstS* ORF and its forward 600-bp fragment was amplified from the 05ZY genome with *tstS*C-1/*tstS*C-2 (Supplementary Table S1). The fragment was cloned into pSET2 to form the knock-in plasmid pSET2-*tstS*.

The knockout mutant Δ*tstS* was constructed by allelic replacement with the plasmid pSET4s-Δ*tstS* using previously described methods (Takamatsu et al., 2001). The complementation and TstS expression strains were generated by electroporating the pSET2-*tstS* plasmid into Δ*tstS* and P1/7. The pSET2 plasmid was electroporated into 05ZY and P1/7 to form the control strains (Smith et al., 1997).

### *In Vivo* Virulence Studies

Sixty female BALB/c mice at the age of 5 weeks were randomly assigned to six groups. Each group was challenged by intraperitoneal injection with 200 μl of strain 05ZY-pSET2, Δ*tstS*-pSET2, or CΔ*tstS* (the complementary strain to the Δ*tstS* mutant strain) at a dose of 4 × 10^8^ CFUs per mouse. The injection dose for each of P1/7-pSET2 and P1/7-*tstS* was 3 × 10^8^ CFUs per mouse. Clinical signs of infection were observed and recorded until the mice recovered or died. The size difference between the mice was ≤1 g.

### Measuring Inflammatory Cytokines and Bacterial Content

One hundred BALB/c mice at the age of 5 weeks were randomly assigned to five groups and injected intraperitoneally with 05ZY-pSET2, Δ*tstS*-pSET2, CΔ*tstS*, P1/7-pSET2, or P1/7-*tstS* at a non-lethal dose (2 × 10^8^ CFUs per mouse). For all groups, five randomly selected mice were sacrificed, dissected, and had peripheral blood taken at 3, 6, 9, and 12 h.p.i. as previously described (Yang et al., 2015a). A 50-μl blood sample was serially diluted and plated onto tryptic soy agar plates to determine the bacterial load. Each sample was assayed using two dilution gradients with three replicates.

Serum was separated from the remaining blood as previously described (Zhang et al., 2015), and the concentrations of TNF-α, IL-6, and IL12p70 in the serum samples were determined using enzyme-linked immunosorbent assay (ELISA) kits in accordance with the manufacturer’s protocols (Dakewe Biotech, Shenzhen, China). All the samples were assayed in triplicate and compared with standards.

### Microarray-Based Comparative Transcriptomic Analysis of ΔtstS

In a previous study, specific 40- to 60-mer oligonucleotide probes were designed to target all 2194 putative ORFs of *S. suis* 05ZYH33 and were printed eight times on the surface of each microarray slide (Agilent, Santa Clara, CA, United States) (Zhang A. et al., 2012). Four biological replicates of 05ZY or Δ*tstS* were prepared and detected as previously described (Zhang A. et al., 2012). Genes with ratio changes greater than 2 and a corrected *P*-value < 0.05 were considered to be differentially expressed genes (Zhang A. et al., 2012). The data discussed in this publication have been deposited in NCBI’s Gene Expression Omnibus (Fontaine et al., 2004) and are accessible through the GEO Series accession number GSE112779^1^.

### Expression and Purification of TstS

The whole coding sequence of *tstS* was amplified using the *tstS*F/*tstS*R primers and cloned into the pET-28a (+) expression vector. The constructed plasmid was transformed into *E. coli* strain BL21 (DE3) for protein expression. TstS was purified using Ni-nitrilotriacetic acid agarose (Bio-Rad Laboratories, Hercules, CA, United States) according to the manufacturer’s instructions and subjected to SDS-polyacrylamide gel electrophoresis analysis.

### Electrophoretic Mobility Shift Assay (EMSA)

The ability of recombinant TstS to bind DNA fragments was detected by EMSA. The 300-bp upstream region from the coding sequence of each gene was amplified and purified as previously described (Zhang T. et al., 2012). Protein and DNA fragments were incubated in 30 μl binding buffer composed of 10 mM 4-(2-hydroxyethyl)-1-piperazineethanesulfonic acid (pH 7.8), 1 mM ethylenediaminetetraacetic acid, 5 mM MgCl_2_, 50 mM KCl, 10% glycerol, 3 μg poly(dI-dC), and 4 μg bovine serum albumin (Shao et al., 2016). Polyacrylamide gels were stained with ethidium bromide after electrophoresis.

### Bactericidal Assays

The bactericidal assays were performed as previously described (Wisselink et al., 1999). Heparinized whole blood was collected from BALB/c mice. The 05ZY-pSET2, Δ*tstS*-pSET2, and CΔ*tstS* strains were harvested at the logarithmic phase of growth, washed twice with phosphate-buffered saline, and diluted to 1 × 10^6^ CFUs/ml. The bacterial suspensions (10 μl) were combined with whole blood (490 μl), complete serum (490 μl), or inactivated serum (490 μl) and the mixtures were rotated at 37°C. Samples were taken at several time points and the number of viable bacteria was determined by plating the samples immediately. We defined the growth factor as the ratio between the number of CFUs in each sample after incubation divided by the number of CFUs at the first-time point (0 h), which was calculated as CFUs_nhours_/CFUs_0 hours_. Heat-inactivated serum was prepared by incubating normal mouse serum at 56°C for 40 min (Schorn et al., 2010).

Polymorphonuclear leukocytes (PMNs) were isolated from the heparinized blood of BALB/c mice by sedimentation in 6% dextran, as previously described (Baltimore et al., 1977). PMN-mediated bacterial killing assays were performed as previously described (Chabot-Roy et al., 2006) with minor modifications. PMNs at a concentration of 8 × 10^6^ cells/ml were mixed with 8 × 10^4^ CFUs/ml of each *S. suis* strain in microtubes in DMEM, centrifuged for 5 s to enhance contact, and then incubated at 37°C with 5% CO_2_. Tubes containing bacteria alone without PMNs were treated similarly and used as controls. Serial dilutions of the mixtures were plated immediately. Colonies were counted and the percentage of SS2 that survived was measured as follows: (CFUs_PMN+_/CFUs_PMN-_) × 100%.

### Ethics Statement

This study was performed in accordance with the recommendations of the Guide for the Care and Use of Laboratory Animals Monitoring Committee of Hubei Province, China, and the protocol was approved by the Committee on the Ethics of Animal Experiments at the College of Veterinary Medicine, Huazhong Agricultural University.

This study was performed in accordance with the recommendations of Good Clinical Practice guidelines. The protocol was approved by the Medical Ethics Committee of the Huazhong Agricultural University Hospital. All subjects gave written informed consent in accordance with the Declaration of Helsinki.

### Statistical Analysis

All assays were repeated at least three times. Data were analyzed by Student’s *t*-test and analysis of variance using the Prism software package (GraphPad Software, La Jolla, CA, United States). *P* < 0.05 was set as the threshold for significance.

## Results

### Identification and Characterization of the Stand-Alone Transcriptional Regulator TstS in the 89K PAI

**FIGURE 1:**
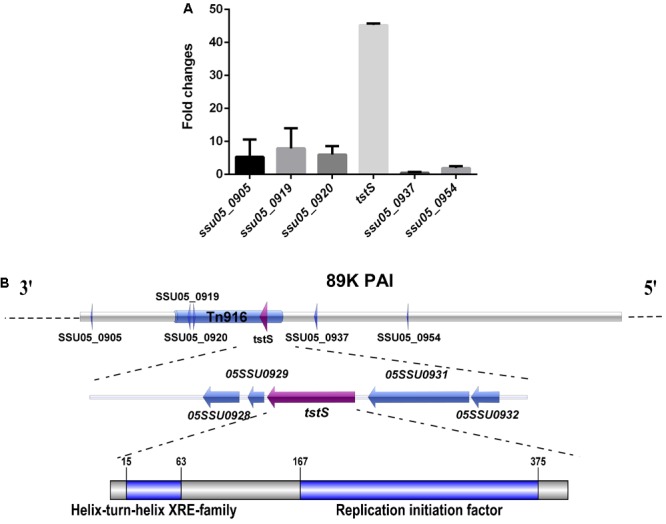
Identification and characterization of the stand-alone transcriptional regulator TstS in the 89K pathogenicity island (PAI). **(A)** *In vivo* expression analysis of six stand-alone transcriptional regulators of the 89K PAI in *Streptococcus suis*. **(B)** The genetic locus of *tstS* within the 89K PAI. The arrow shows the direction of transcription and the components were drawn to scale using the Illustrator for Biological Sequences software (Liu et al., 2015).

Six stand-alone transcriptional regulators were found in the 89K PAI via gene annotation and the BLASTn program. The transcript abundances of these six genes *in vivo* were studied, and *tstS* was the most highly expressed (**Figure 1A**).

The transcriptional regulator TstS was found in *Tn916*, which is located on the 89K PAI, suggesting a possible relationship with STSLS. The conserved domains were analyzed via the BLASTp program. This protein was found to be highly conserved among different species such as *Staphylococcus, Streptococcus, Lactobacillus*, and *Facklamia* (Supplementary Figure S1). TstS contains a helix-turn-helix DNA-binding domain at the N-terminus, which is a common feature among members of the xenobiotic response element family (Barragan et al., 2005; Santos et al., 2009). A replication initiation factor domain was also discovered at its C-terminus, indicating topoisomerase activity during replication, recombination, and repair (**Figure 1B**).

The genetic structures of *tstS* and its flanking genes were also defined. No putative promoter could be identified at the 300-bp upstream region based on an analysis of the TstS coding sequence using the Soft Berry BPROM software (Jacobs et al., 1996). SSU05_0929, which is situated downstream of the *tstS* gene, encodes a 73-aa polypeptide for which there were no putative conserved domain matches. The SSU05_0928 gene was predicted to be an antirestriction protein that allows the unmodified plasmid to evade restriction in the recipient bacterium. The function of the upstream gene SSU05_0932 was still unknown, while SSU05_0931 was recognized to function as a DNA translocase. *tstS* and its flanking genes were predicted to not lie in the same operon by the MicrobesOnline Operon Predictions software (Wisselink et al., 2001).

### *tstS* Is Upregulated *in Vivo* and in Human Blood

**FIGURE 2:**
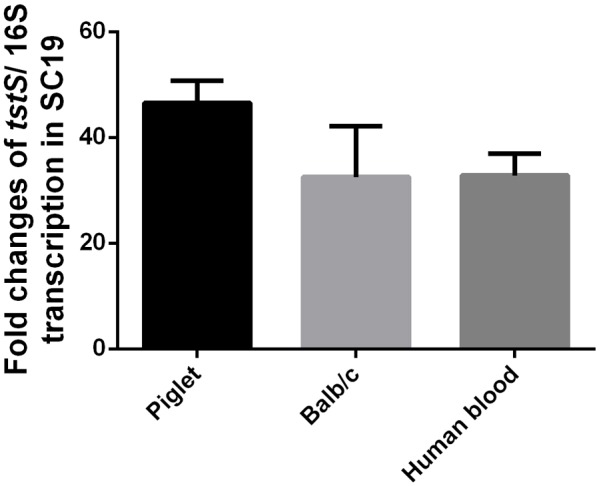
Expression of TstS detected by quantitative polymerase chain reaction (qPCR) *in vivo* and in human blood. RNA samples from *S. suis* were obtained from *in vitro* bacterial cultures, piglet, and mouse blood when hosts presented typical symptoms of *S. suis* infection, and human blood. The bars represent the standard error of the mean, based on three independent experiments.

Quantitative PCR was used to compare the expression changes of *tstS in vivo* and *in vitro*. *S. suis* RNA was obtained from *in vitro* bacterial cultures, piglet or mouse blood when the hosts presented typical symptoms of *S. suis* infection, and human blood. *tstS* expression was increased in the two animal models and human blood as compared with its expression in the *in vitro* cultures (**Figure 2**).

### *tstS*-Expressing Strains Stimulate Higher Levels of Cytokine Release From Macrophages

To evaluate the role of TstS in 05ZY pathogenesis, we constructed a knockout mutant, Δ*tstS*, and its complementary strain, CΔ*tstS*. An empty pSET2 plasmid was electroporated into the 05ZY and Δ*tstS* strains to eliminate other influencing factors and the resulting new strains were named 05ZY-pSET2 and Δ*tstS*-pSET2, respectively (Supplementary Figure S2A). Δ*tstS*-pSET2 and 05ZY-pSET2 did not differ in their growth behaviors (Supplementary Figure S2B).

**FIGURE 3:**
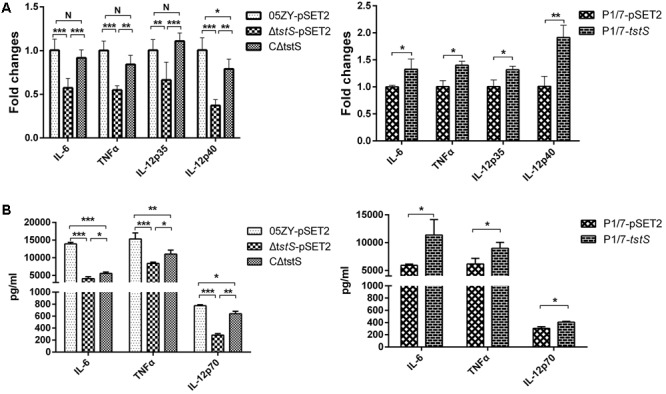
Cytokines from macrophages were analyzed by qPCR and enzyme-linked immunosorbent assay. Macrophages were stimulated with the strains 05ZY-pSET2, Δ*tstS*-pSET2, CΔ*tstS*, P1/7-pSET2, or P1/7-*tstS* at the dose of 5 × 10^6^ CFUs per well for 6 h and then analyzed by qPCR and enzyme-linked immunosorbent assay. **(A)** The mRNA levels of TNF-α, IL-6, IL-12p35, and IL-12p40 were examined by qPCR. **(B)** The protein concentrations of cytokines in the culture supernatants were examined by enzyme-linked immunosorbent assay. The bars represent the standard error of the mean, based on three independent experiments. ^∗∗∗^*P* < 0.001; ^∗∗^*P* < 0.01; ^∗^*P* < 0.05.

Next, we investigated the proinflammatory activity of the constructed strains in macrophages. After the macrophages were incubated with the 05ZY-pSET2, Δ*tstS*-pSET2, or CΔ*tstS* strain, the protein and transcript abundances of TNF-α, IL-6, and IL-12p70 were measured by ELISA and qPCR, respectively. According to the qPCR analysis, IL-12p35 and IL-12p40 were detected instead of IL-12p70 (Abdi et al., 2014). 05ZY-pSET2 and CΔ*tstS* induced higher levels of TNF-α, IL-6, IL-12p35, and IL-12p40 mRNA expression than Δ*tstS*-pSET2 did (**Figure 3A**). The ELISA analysis detected higher levels of TNF-α, IL-6, and IL-12p70 protein secretion in the 05ZY-pSET2-infected and CΔ*tstS*-infected groups (**Figure 3B**).

The P1/7 strain, which does not cause STSLS, was used to construct the TstS expression strain (P1/7-*tstS*) and its control strain (P1/7-pSET2) (Supplementary Figure S2A). Similar to 05ZY and CΔ*tstS*, P1/7-*tstS* stimulated the expression of IL-6 and IL-12p70 more strongly than P1/7-pSET2 did (*P* < 0.05) (**Figure 3B**).

### TstS Contributes to SS2 Virulence in Mice

**FIGURE 4:**
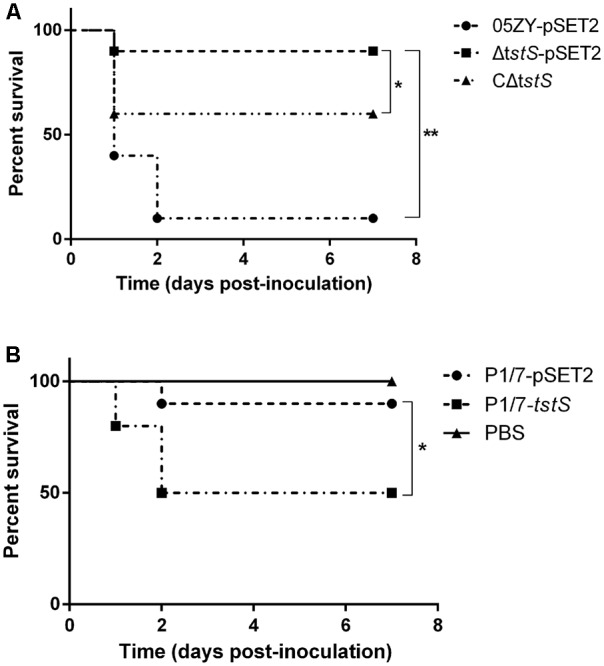
Survival experiments in BALB/c mice. **(A)** Thirty female BALB/c mice at the age of 5 weeks were randomly assigned to three groups. Mice were intraperitoneally infected with 05ZY-pSET2 (●), Δ*tstS*-pSET2 (■), or CΔ*tstS* (▲) at a dose of 4 × 10^8^ CFUs per mouse. ^∗∗^*P* < 0.01 for 05ZY-pSET2 versus Δ*tstS*-pSET2 and ^∗^*P* < 0.05 for Δ*tstS*-pSET2 versus CΔ*tstS*. **(B)** Thirty female BALB/c mice at the age of 5 weeks were randomly assigned to three groups equally. The P1/7-pSET2 (▼) and P1/7-*tstS* (♦) strains were also intraperitoneally administered at a dose of 3 × 10^8^ CFUs per mouse. Animals in the control group were intraperitoneally injected with phosphate-buffered saline (▼). ^∗^*P* < 0.05 for P1/7-pSET2 versus P1/7-*tstS* CΔ*tstS*. The results shown are representative of three independent experiments.

To investigate the contribution of TstS to the virulence of 05ZY, independent experimental infections with lethal doses of 05ZY-pSET2, Δ*tstS*-pSET2, or CΔ*tstS* were administered to BALB/c mice (4 × 10^8^ CFUs per mouse). After 6 h post-inoculation, all the mice challenged with 05ZY or CΔ*tstS* developed typical clinical symptoms including a rough coat, lethargy, and conjunctivitis. After 1 day post-inoculation, one of the remaining 05ZY-infected mice showed nervous signs such as walking in circles, while the others exhibited obnubilation, which was considered to be STSLS (Benga et al., 2004). Mice infected with Δ*tstS* showed mild clinical signs. Ninety percent of the 05ZY-infected mice died within 2 days post-inoculation (d.p.i.), 40% of the CΔ*tstS*-infected mice died within 1 d.p.i., and only one Δ*tstS*-infected mouse died (**Figure 4A**). The Δ*tstS* strain was less virulent than both the 05ZY and CΔ*tstS* strains (*P* < 0.01 and *P* < 0.05, respectively; **Figure 4A**).

Although the P1/7 strain has not been reported to cause acute death, it still shows virulence in mice. An injection dose of 3 × 10^8^ CFUs per mouse was chosen for the P1/7-pSET2 and P1/7-*tstS* strains. All the mice challenged with P1/7-*tstS* developed typical clinical symptoms similar to those of 05ZY infection: 60% of the mice exhibited lethargy at 1 d.p.i., with a more acute onset than the P1/7-pSET2-infected group. Half of the P1/7-*tstS*-infected mice died within 2 d.p.i., whereas only 10% of the P1/7-pSET2-infected mice died within 2 d.p.i. (**Figure 4B**).

### TstS Stimulates Bacteremia

**FIGURE 5:**
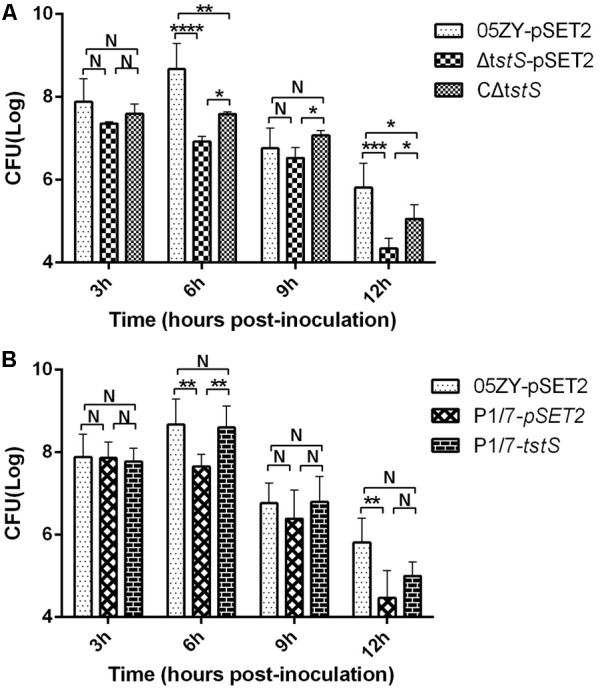
Bacterial counts. **(A)** Mice were infected with 05ZY-pSET2, Δ*tstS*-pSET2, or CΔ*tstS* at a dose of 2 × 10^8^ CFUs per mouse. Five randomly selected mice were used for bacterial load analysis. ^∗∗∗∗^*P* < 0.0001; ^∗∗∗^*P* < 0.001; ^∗∗^*P* < 0.01; ^∗^*P* < 0.05. **(B)** The bacterial loads of P1/7-pSET2 and P1/7-*tstS* were also measured and compared with those of the 05ZY-pSET2 group. ^∗∗^*P* < 0.01 for P1/7-*tstS* versus P1/7-pSET2 at 6 h post-inoculation. The bars represent the standard error of the mean, based on three independent experiments.

To assess the role of TstS in bacteremia, independent experimental infections with 05ZY, Δ*tstS*, or CΔ*tstS* were performed in BALB/c mice. These strains produced similar bacterial loads at 3 h.p.i., but between 3 and 6 h.p.i. the bacterial loads increased in the 05ZY-pSET2-infected group (6.1 times) and CΔ*tstS*-infected group (1.05 times) while the bacterial loads of the Δ*tstS*-infected group decreased by 63% (**Figure 5A**). We also evaluated the ability of TstS to cause bacteremia in P1/7 in BALB/c mice. The bacterial loads of the P1/7-pSET2 and P1/7-*tstS* infected groups exhibited similar kinetics, but that of the P1/7-*tstS*-infected group was remarkably higher at 6 h.p.i. (*P* < 0.01) (**Figure 5B**).

### TstS Increases Cytokine Production

**FIGURE 6:**
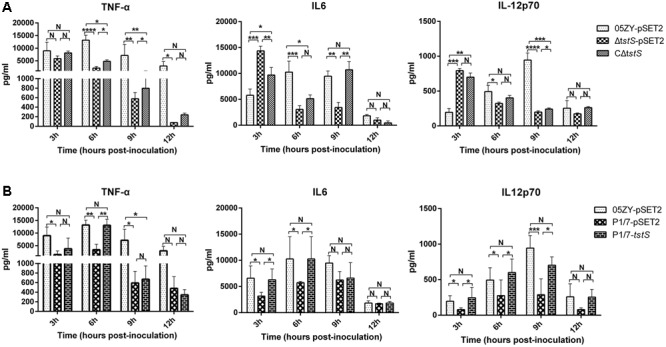
Serum levels of TNF-α, IL-6, and IL-12p70 in BALB/c mice infected with each strain. Five randomly selected mice per group were examined. **(A)** Mice were infected with 05ZY-pSET2, Δ*tstS*-pSET2, or CΔ*tstS* at a dose of 2 × 10^8^ CFUs per mouse. ^∗∗∗∗^*P* < 0.0001; ^∗∗∗^*P* < 0.001; ^∗∗^*P* < 0.01; ^∗^*P* < 0.05. **(B)** Mice were infected with P1/7-pSET2 or P1/7-*tstS* at a dose of 2 × 10^8^ CFUs per mouse. Data were compared with those from the 05ZY-pSET2 strain. The bars represent the standard errors of the means, based on three independent experiments. ^∗∗∗^*P* < 0.001; ^∗∗^*P* < 0.01; ^∗^*P* < 0.05.

The serum levels of TNF-α, IL-6, and IL-12p70 were measured at 3, 6, 9, and 12 h.p.i. (**Figure 6**). The levels of all three cytokines were higher in the 05ZY-pSET2-infected group than in the Δ*tstS*-pSET2-infected group during the acute phase (mainly at 6 and 9 h.p.i.). The Δ*tstS*-infected group produced more IL-6 and IL-12p70 at 3 h.p.i. than the 05ZY-pSET2-infected or CΔ*tstS*-infected groups. The CΔ*tstS*-infected group stimulated a higher release of these cytokines and especially TNF-α during the acute phase (at 6 and 9 h.p.i.).

The ability of TstS to stimulate the release of TNF-α, IL-6, and IL-12p70 was also observed in the P1/7 strain. As expected, P1/7-pSET2 induced lower levels of inflammatory cytokine production than 05ZY, whereas P1/7-*tstS* induced higher levels. Our results suggested that TstS promotes the secretion of proinflammatory cytokines that are involved in the development of STSLS.

### TstS Influences Multiple Virulence- and Metabolism-Related Genes

**FIGURE 7:**
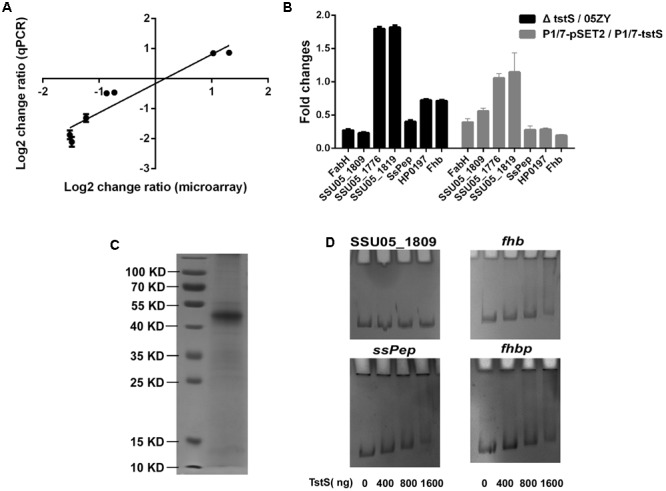
TstS influences multiple virulence- and metabolism-related genes. **(A)** Seven representative genes were selected to determine the correlation between the fold changes (Δ*tstS* vs. 05ZY) from the microarray and qPCR data. The linear equation was expressed as: *Y* = 0.97*X* - 0.1615088 (*R*^2^ = 0.9064). **(B)** The column shows the fold changes (Δ*tstS* vs. 05ZY or P1/7-pSET2 vs. P1/7-*tstS*) of the seven indicated genes. The primers used for qPCR are listed in Supplementary Table S1. **(C)** Representative SDS-polyacrylamide gel electrophoresis analysis of purified recombinant TstS. **(D)** TstS was observed to bind to the SsPep, Fhbp, and Fhb promoters (300 bp) at different concentrations. The SSU05_1809 promoter (300 bp) was used as the negative control.

To identify the genes affected by *tstS*, the transcriptomic profile of Δ*tstS* was compared with that of the wild-type strain using an SS2 genomic microarray (Supplementary Table S2). The results were validated by qPCR and a strong positive correlation was observed between the two methods (**Figure 7A**). The transcript abundances of 33 genes were observed to significantly differ between these strains (change ratio ≥ 2, *P* ≤ 0.05). Of these 33 genes, the transcript abundances of 29 genes were decreased in Δ*tstS* as compared with the wild-type strain and those of four genes were increased. Among the genes whose transcript abundances were decreased in Δ*tstS*, 75.9% (22 of 29) were predicted to be involved in metabolism, of which 72.7% (16 of 22) were directly related to fatty acids metabolism. The transcript abundance of the virulence-related gene that encodes the protein SsPep was also decreased (Tan et al., 2011). Among the genes with increased levels, 50% (2 of 4) were glycometabolism-related genes, and none were virulence-related.

The transcript abundances of 56 genes were observed to show smaller changes (change ratio ≥ 1.5, *P* ≤ 0.05). Of these 56 genes, the transcript abundances of 27 genes were decreased in *ΔtstS* as compared with that in the wild-type strain and those of 29 genes were increased. Of the genes with increased mRNA levels, 41.3% (12 of 29) of them were glycometabolism-related genes and several were involved in the sugar ABC transporter system or PTS system. Of the genes with decreased mRNA levels, 22.2% (6 of 27) were predicted to be involved in metabolism, and 22.2% (6 of 27) were predicted to be cell wall proteins or membrane proteins. Several virulence-related genes were found to be controlled by TstS, including immune evasion-related genes (*fhbp* and *fhb*) and adherence-related genes (SSU05_2103), and this was verified in the P1/7 strain (**Figure 7B**). TstS influences multiple virulence- and metabolism-related genes, which may explain its pathogenicity.

The results of the EMSA analysis showed that the recombinant TstS could bind to the promoters of SsPep, Fhbp, and Fhb, but not to the promoters of metabolism-related genes (the SSU05_1809 promoter was used as the negative control) (**Figures 7C,D**). The original EMSA pictures are shown in Supplementary Figure S3. These results suggested that TstS can directly regulate SsPep, Fhbp, and Fhb.

### TstS Facilitates the Growth of *S. suis* in the Blood and Increases the Resistance of SS2 Against PMN-Mediated Bacterial Killing

**FIGURE 8:**
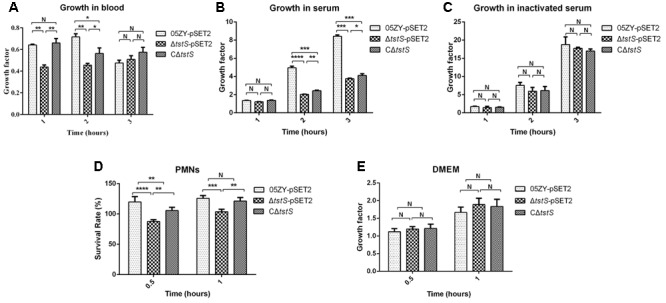
TstS increases the resistance of SS2 to phagocytosis and facilitates the growth of *S. suis* in blood. Each strain was diluted to 1 × 10^6^ CFUs/ml. Aliquots of the bacterial suspensions (10 μl) were combined with whole blood, complete serum, or inactivated serum (490 μl), and the mixtures were rotated at 37°C. We defined the growth factor as the ratio between the number of CFUs in each sample after incubation divided by the number of CFUs at the first time point (0 h), which was calculated as CFUs_nhours_/CFUs_0 hours_. **(A)** The growth of each strain in mouse blood. **(B)** The growth of each strain in complete mouse serum. **(C)** The growth of each strain in inactivated mouse serum. **(D)** The survival of each strain in the PMN-mediated bacterial killing assay. The percentage of SS2 that survived was measured as follows: (CFUs_PMN+_/CFUs_PMN-_) × 100%. **(E)** The growth of each strain in Dulbecco’s modified Eagle’s medium (DMEM). The bars represent the standard error of the mean, based on three independent experiments. ^∗∗∗^*P* < 0.001; ^∗∗^*P* < 0.01; ^∗^*P* < 0.05.

To determine the role of TstS in the evasion of innate immune responses, we measured the growth of the 05ZY-pSET2, Δ*tstS*-pSET2, and CΔ*tstS* strains in whole blood, complete serum, or inactivated serum from mice. In whole blood, the mean growth factors of Δ*tstS*-pSET2 after 1, 2, and 3 h of incubation were 0.611, 1.098, and 1.268, while those of 05ZY-pSET2 were 0.905, 2.103, and 4.038, respectively. Although the growth factor of CΔ*tstS* did not reach the level of 05ZY-pSET2, it was still significantly higher than that of Δ*tstS*-pSET2 (**Figure 8A**). In complete serum, the mean growth factors of each strain showed similar trends to those observed in whole blood, although the cell number of each strain did not decrease after 1 h of incubation (**Figure 8B**). In inactivated serum, there was no significant difference among the three groups (**Figure 8C**). These results suggested that TstS increases the resistance of SS2 to heat-labile serum factors and facilitates the growth of *S. suis* in the blood.

Finally, we evaluated the role of TstS in PMN-mediated bacterial killing. The results showed that the Δ*tstS*-pSET2 group exhibited a significantly lower survival rate as compared with the 05ZY group following incubation for 0.5 h (*P* < 0.0001) and 1 h (*P* = 0.004), respectively (**Figure 8D**), while in DMEM the growth factors of these strains did not significantly differ (**Figure 8E**). These findings suggested that a lack of TstS increases the vulnerability of *S. suis* to being killed by the PMNs.

## Discussion

Until the first human infection was reported in 1968, *S. suis* was considered to be a pathogen of the swine only (Perch et al., 1968). There were 1,642 cases of *S. suis* infections identified between 2002 and 2013 worldwide in humans, and the infections resulted in meningitis, septicemia, and arthritis (Benga et al., 2004). Two large-scale outbreaks in humans showed that some Chinese SS2 strains can also cause serious STSLS (Tang et al., 2006; Chen et al., 2007). Infections with these strains are characterized by an acutely high fever, vascular collapse, hypotension, shock, multiple organ failure, and ultimately death (Tang et al., 2006; Chen et al., 2007). Cytokine levels were higher in the serum samples from patients with STSLS than in those from patients with meningitis only (Ye et al., 2009). An assessment of the pathogenesis of SS2 infection in piglets showed that acute and persistent bacteremia and systemic cytokine storms are directly and closely related to the progression of the disease into a severe form that is manifested as septic shock and STSLS (Bi et al., 2014).

In general, streptococcal toxic shock syndrome (STSS) is associated with strains that produce bacterial superantigens such as SpeA, SpeC, and SSA (Stevens and Bryant, 2016). However, no putative superantigen or homologous gene was identified in the genomes of SS2 isolates associated with STSLS, indicating that several unique mechanisms could be involved (Zhang et al., 2008, 2017). Through a comparative genomics analysis, a pathogenicity island with a size of 89 kb was found to exist only in the Chinese strains of SS2. This feature was named the 89K PAI, and its presence was considered to be related to the ability to cause STSLS (Chen et al., 2007).

In this study, a gene named *tstS* in the 89K PAI, which encodes the transcriptional factor TstS, was found to be highly expressed *in vivo*. The activation of *tstS* during infection suggests that it contributes to bacterial pathogenesis. In addition, the increased transcript abundance of *tstS* in human blood implied that its expression may also increase during human infection. The Δ*tstS* mutant strain stimulated cytokines to a lesser extent than the 05ZY strain in macrophages at 6 h.p.i. (**Figure 3A**).

Bacteremia and high cytokine levels promote the high fatality rate of STSLS (Chu et al., 2006; Bi et al., 2014; Yang et al., 2015b). In previous studies, STSLS induced high cytokine levels and bacterial loads during the acute phase, usually within 6–12 h.p.i., at a non-lethal dose in the host (Zhao et al., 2011; Bi et al., 2014; Eisenberg et al., 2015). Here, bacteremia and cytokine expression were evaluated in a mouse model with the use of 05ZY as an STSLS-positive strain. As expected, the 05ZY-pSET2-infected group exhibited higher levels of bacteremia and cytokine expression than the Δ*tstS*-infected group during the acute phase. This suggests that TstS promotes the secretion of proinflammatory cytokines that are involved in the development of STSLS.

Although the complementary strain CΔ*tstS* did not fully restore the wild-type phenotype, bacterial loads and cytokine levels were higher than the Δ*tstS*-infected group at 6 h.p.i. One possible reason that CΔ*tstS* did not fully restore the wild-type phenotype is that the complementary plasmid was partly lost without antibiotic pressure *in vivo* (Smith et al., 1997; Takamatsu et al., 2001). To mimic horizontal gene transfer, the virulent European strain P1/7 was used, since this strain does not contain the *tstS* gene and cannot cause STSLS (Zhao et al., 2011; Wu et al., 2014). The complementary plasmid containing the *tstS* gene was transformed into P1/7 to construct a TstS expression strain, P1/7-*tstS*. P1/7-*tstS* was more virulent than P1/7-pSET2 and reached higher bacteremia level at 6 h.p.i. in mice, which was similar to the 05ZY strain (**Figure 5B**). P1/7-*tstS* also induced higher levels of cytokine expression in the host (**Figure 6B**). Together, these data indicate that *S. suis* strains containing *tstS* cause high levels of bacteremia and cytokine release in the blood, and these parameters are related to STSLS.

High levels of IL-6 and IL-12p70 expression were stimulated by Δ*tstS* at early stages, and these cytokines are believed to contribute to efficient bacterial clearance (Murphey et al., 2004; Stoycheva and Murdjeva, 2005). This increased cytokine production may be attributed to the loss of two TstS-regulated proteins known as Fhb and Fhbp, which can block the alternative pathway of the complement system during the early stages of infection and inhibit cytokine production (Pian et al., 2012; Vaillancourt et al., 2013).

A transcriptomics analysis was used to compare global transcription between the 05ZY strain and the Δ*tstS* strain. Most of the differentially expressed genes were located outside of the 89K PAI. The transcript abundance of *fhb* decreased when *tstS* was knocked out. Fhb can bind factor H and C3b/C3d on the bacterial surface to form a large immune complex. This immune complex contributes to the evasion of PML-mediated phagocytic clearance, which is central to the establishment of bacteremia caused by SS2 (Pian et al., 2012; Li et al., 2016). The transcript abundance of *fhbp* was also decreased in Δ*tstS*, which is likely related to immune evasion via binding to factor H (Vaillancourt et al., 2013). In addition, Fhbp was reported to influence the expression of glycometabolism-related genes, which is likely related to the formation of CPS in SS2 (Zhang A. et al., 2012). CPS is one of the most important virulence genes in *S. suis* (Zhang A. et al., 2012) and it is considered to be responsible for inducing the release of cytokines (Calzas et al., 2013; Zhang et al., 2015, 2017). We also observed a decreased transcript abundance of fatty acid biosynthesis genes, which may result in the decreased fatty acid content in the Δ*tstS* strain. The lack of fatty acids would diminish cell membrane integrity and thereby reduce virulence (Kuipers et al., 2015; Yao and Rock, 2015). Although multiple metabolism-associated factors were found in the results, Δ*tstS* did not show an altered growth behavior in culture medium or heat-inactivated serum as compared with the wild-type strain.

Here, homologous genes of *tstS* were identified by searching its sequence against bacterial protein databases. Highly conserved homologs of TstS were found in species from several bacterial genera including *Staphylococcus, Streptococcus, Lactobacillus*, and *Facklamia* (Supplementary Figure S1). All the TstS homologs were observed to be located on a *Tn916* transposon, including that of the 05ZY strain of *S. suis* (**Figure 1**). The Tn916 family is a group of mobile genetic elements that are widespread among many commensal and pathogenic bacteria (Roberts and Mullany, 2009). In this paper, the P1/7-*tstS* strain caused high levels of bacteremia and cytokine release similar to those induced by the 05ZY strain, indicating that *S. suis* can acquire the ability to cause STSLS from other bacteria by transposon mutagenesis.

## Conclusion

We identified an *in vivo*-induced transcriptional factor, TstS, that promotes SS2 pathogenesis. Further analyses revealed that the strain containing *tstS* stimulated higher levels of cytokine production and bacteremia in the host, and infection with this strain negatively affected the survival of the infected animals. A transcriptomics analysis confirmed that TstS regulates genes related to metabolism and immune invasion.

## Author Contributions

ZX and BC conceived and designed the study. ZX, QZ, LL, YY, KH, SY, and JY performed the experiments. XS work on reagent preparation. ZX and MJ wrote the paper. BC, QZ, and AZ reviewed and edited the manuscript. All authors read and approved the manuscript.

## Conflict of Interest Statement

The authors declare that the research was conducted in the absence of any commercial or financial relationships that could be construed as a potential conflict of interest.

## Footnotes

1 https://www.ncbi.nlm.nih.gov/geo/query/acc.cgi?acc=GSE112779
