# Supplementary material for: Streptococcus suis 2 Transcriptional Regulator TstS Stimulates Cytokine Production and Bacteremia to Promote Streptococcal Toxic Shock-Like Syndrome
Source: Front Microbiol. 2018 Jun 19;9:1309. doi: 10.3389/fmicb.2018.01309 (PMC6020791; doi:10.3389/fmicb.2018.01309)
Supplement: Supplementary file 1 [file Table_1.doc]

**Table S1. Expression levels of genes in ΔtstS compared to WT confirmed by microarray analysis.**

| Code for ORF | Fold changes  (WT/Δ*tstS*) | Corrected  p-value | Functional annotation |  |
| --- | --- | --- | --- | --- |
| SSU05_0154 | 0.400391581 | 8.43E-05 | hypothetical protein SSU05_0154 | + |
| SSU05_0153 | 0.425869624 | 9.49E-05 | Predicted metalloendopeptidase | + |
| SSU05_1663 | 0.47898465 | 4.11E-05 | Methyl-accepting chemotaxis protein | + |
| SSU05_1776 | 2.034898337 | 0.011898392 | Permease | + |
| SSU05_1794 | 0.40533036 | 0.035069776 | Histone acetyltransferase HPA2 | + |
| SSU05_1795 | 0.287980063 | 0.022158026 | Histone acetyltransferase HPA2 | + |
| SSU05_1796 | 0.066535556 | 0.001416296 | Acetyl-CoA carboxylase alpha subunit | + |
| SSU05_1797 | 0.053753933 | 0.000976687 | Acetyl-CoA carboxylase beta subunit | + |
| SSU05_1798 | 0.053330288 | 0.001066231 | Acetyl-CoA carboxylase beta subunit | + |
| SSU05_1799 | 0.051395849 | 0.001046967 | Biotin carboxylase | + |
| SSU05_1800 | 0.044038852 | 0.000992103 | 3-hydroxymyristoyl/3-hydroxydecanoyl-(acyl carrier protein) dehydratase | + |
| SSU05_1801 | 0.043664482 | 0.001100784 | Biotin carboxyl carrier protein | + |
| SSU05_1802 | 0.045588435 | 0.000636728 | 3-oxoacyl-(acyl-carrier-protein) synthase | + |
| SSU05_1803 | 0.048631556 | 0.000912638 | Dehydrogenase | + |
| SSU05_1804 | 0.065296393 | 0.000738484 | Malonyl CoA-acyl carrier protein transacylase | + |
| SSU05_1805 | 0.140541251 | 0.003384108 | Dioxygenase | + |
| SSU05_1806 | 0.381248407 | 0.01056884 | Acyl carrier protein | + |
| SSU05_1807 | 0.348151842 | 0.013310305 | 3-oxoacyl-[acyl-carrier-protein] synthase | + |
| SSU05_1808 | 0.390479774 | 0.010199451 | Transcriptional regulator | + |
| SSU05_1809 | 0.357180734 | 0.008555992 | Enoyl-CoA hydratase/carnithine racemase | + |
| SSU05_1815 | 0.435001693 | 1.58E-07 | Transcriptional regulator/sugar kinase | + |
| SSU05_1817 | 3.134449041 | 0.000336814 | Phosphotransferase system IIC component | + |
| SSU05_1818 | 2.697686385 | 0.00010232 | Beta-fructosidases | + |
| SSU05_1819 | 2.477067543 | 0.00134125 | Transcriptional regulator | + |
| SSU05_0389 | 0.223696752 | 0.000105206 | ATPases | + |
| SSU05_0390 | 0.224508595 | 8.26E-05 | ATPases | + |
| SSU05_0391 | 0.251344774 | 0.0001812 | ATPases | + |
| SSU05_0509 | 0.324697376 | 0.03545022 | Sugar kinase | + |
| SSU05_0625 | 0.493543804 | 0.012289971 | Histone acetyltransferase HPA2 | + |
| SSU05_0627 | 0.371769169 | 0.006884709 | Carbamate kinase | + |
| SSU05_0628 | 0.343932081 | 0.001995265 | Predicted membrane protein | + |
| SSU05_0689 | 0.352769911 | 0.000853635 | Phosphopantothenoylcysteine synthetase/decarboxylase | + |
| SSU05_0815 | 0.398591135 | 0.007476656 | GMP reductase | + |
| SSU05_1664 | 0.507725 | 0.001716 | hypothetical protein SSU05_1664 |  |
| SSU05_0624 | 0.510596 | 0.022113 | arginine deiminase |  |
| SSU05_1371 | 0.519111 | 0.002896 | Ribonucleases G and E |  |
| SSU05_0274 | 0.522507 | 0.000428 | Methyl-accepting chemotaxis protein |  |
| SSU05_1348 | 0.525085 | 0.017142 | Cation transport ATPase |  |
| SSU05_2127 | 0.532766 | 0.001299 | Surface antigen |  |
| SSU05_0298 | 0.539169 | 0.00441 | Transcriptional regulator of heat shock gene |  |
| SSU05_0272 | 0.548288 | 0.002399 | Fhb |  |
| SSU05_0329 | 0.559379 | 0.001857 | Unknown protein |  |
| SSU05_0863 | 0.568877 | 0.00031 | hypothetical protein SSU05_0863 |  |
| SSU05_2103 | 0.572597 | 0.004474 | cell wall anchor protein |  |
| SSU05_2145 | 0.5803 | 0.026652 | glucocerebrosidase |  |
| SSU05_0833 | 0.583916 | 0.037884 | hypothetical protein SSU05_0833 |  |
| SSU05_1610 | 0.587226 | 0.007496 | hypothetical protein SSU05_1610 |  |
| SSU05_0688 | 0.588116 | 0.009416 | Phosphopantothenoylcysteine synthetase |  |
| SSU05_0405 | 0.591843 | 0.000327 | hypothetical protein SSU05_0405 |  |
| SSU05_0196 | 0.602876 | 0.000404 | Fhbp |  |
| SSU05_0275 | 0.612576 | 0.001626 | hypothetical protein SSU05_0275 |  |
| SSU05_0862 | 0.626909 | 0.001444 | site-specific recombinase |  |
| SSU05_0636 | 0.6307 | 0.002348 | hypothetical protein SSU05_0636 |  |
| SSU05_0864 | 0.635776 | 0.003885 | putative replication initiator protein |  |
| SSU05_0299 | 0.636901 | 0.006714 | Molecular chaperone GrpE |  |
| SSU05_1233 | 0.639867 | 0.004741 | probable surface antigen |  |
| SSU05_0865 | 0.641983 | 0.034196 | hypothetical protein SSU05_0865 |  |
| SSU05_2100 | 0.64605 | 0.002167 | Unknown protein |  |
| SSU05_0756 | 0.657638 | 0.01063 | Predicted membrane protein |  |
| SSU05_1719 | 0.665705 | 5.98E-05 | Histone acetyltransferase HPA2 |  |
| SSU05_1752 | 1.507749 | 0.000583 | prenyltransferases |  |
| SSU05_1366 | 1.520525 | 0.043331 | Threonyl-tRNA synthetase |  |
| SSU05_1365 | 1.525895 | 0.015904 | permease |  |
| SSU05_2183 | 1.527767 | 0.002446 | inosine-5'-monophosphate dehydrogenase |  |
| SSU05_1361 | 1.530556 | 0.003163 | ATPase |  |
| SSU05_1430 | 1.532742 | 0.009246 | Large-conductance mechanosensitive channel |  |
| SSU05_0035 | 1.533289 | 0.019596 | Phosphoribosylaminoimidazole carboxylase |  |
| SSU05_0525 | 1.539258 | 0.008637 | sugar ABC transporter substrate-binding protein |  |
| SSU05_1040 | 1.544666 | 0.009057 | Tagatose-1,6-bisphosphate aldolase |  |
| SSU05_0714 | 1.547985 | 0.007553 | Pyruvate-formate lyase |  |
| SSU05_1523 | 1.56461 | 0.023215 | hypothetical protein SSU05_1523 |  |
| SSU05_0715 | 1.584402 | 0.001451 | Transaldolase |  |
| SSU05_0991 | 1.586475 | 0.021141 | Rhodanese-related sulfurtransferase |  |
| SSU05_1042 | 1.595247 | 0.011814 | Ribose 5-phosphate isomerase RpiB |  |
| SSU05_0932 | 1.597936 | 0.014899 | hypothetical protein SSU05_0932 |  |
| SSU05_1486 | 1.598448 | 6.50E-05 | permease |  |
| SSU05_1045 | 1.599872 | 0.007639 | Transcriptional regulator of sugar metabolism |  |
| SSU05_1526 | 1.600504 | 0.003526 | Predicted transcriptional regulator |  |
| SSU05_0033 | 1.613698 | 0.005347 | Phosphoribosylamine-glycine ligase |  |
| SSU05_1038 | 1.631175 | 0.022306 | Phosphotransferase system cellobiose-specific component IIA |  |
| SSU05_0034 | 1.658985 | 0.004432 | Phosphoribosylcarboxyaminoimidazole (NCAIR) mutase |  |
| SSU05_0301 | 1.685692 | 0.000306 | hypothetical protein SSU05_0301 |  |
| SSU05_0993 | 1.694558 | 0.015067 | hypothetical protein SSU05_0993 |  |
| SSU05_0302 | 1.714309 | 0.000967 | DnaJ-class molecular chaperone |  |
| SSU05_1484 | 1.738784 | 0.010953 | ATPase |  |
| SSU05_1515 | 1.739915 | 0.000277 | hypothetical protein SSU05_1515 |  |
| SSU05_1487 | 1.766539 | 0.000794 | ABC-type uncharacterized transport system, periplasmic component |  |
| SSU05_1524 | 1.779127 | 0.008335 | hypothetical protein SSU05_1524 |  |
| SSU05_1485 | 1.781914 | 0.002526 | ATPase |  |

*In this column, “+” indicated that the change ratio of these genes is above 2.
