# Supplementary material for: Streptococcus suis 2 Transcriptional Regulator TstS Stimulates Cytokine Production and Bacteremia to Promote Streptococcal Toxic Shock-Like Syndrome
Source: Front Microbiol. 2018 Jun 19;9:1309. doi: 10.3389/fmicb.2018.01309 (PMC6020791; doi:10.3389/fmicb.2018.01309)
Supplement: Supplementary file 2 [file Table_2.docx]

**Table S2. Primer sequences used for this study**

| Gene | Primer sequence |
| --- | --- |
| tstSL-1 | Forward: 5- ATTGAATTCGATACCATTTCCCATAGTCGA (EcoRI) |
| tstSL-2 | Reverse: 5- CCATGGATCCGGACAAAACCTTTATAACTGG (BamHI) |
| tstSR-1 | Forward: 5- TAAGTCGACGCCCATTTGACATACGCTGA (Sal I) |
| tstSR-2 | Reverse: 5- AAGAAGCTTTGATGAATACGTCGCTTTCATGG (Hind III) |
| SPC-1 | Forward: 5- ATCGACTAGTGTTCGTGAATACATGTTATA |
| SPC-2  tstS-1  tstS-2 | Reverse: 5- GCAAGGGACTAGTGTTTTCTAAAATCTGAT  Forward: 5- GAATGAACAAACTTGGTTACAGC  Reverse: 5- TCACGTCCTGTTCTTTTACTGAC |
| tstSC-1 | Forward: 5- CGCGCATGCGGAAATGCTGGGAACAAAAG (SphI) |
| tstSC-2 | Reverse: 5- AAAGGATCCTTATTTTTTTGTTGTTATCACGTCC (BamHI) |
| FabHrt-1 | Forward: 5- GGAGACCAGTGATGAGTGGATTC |
| FabHrt-2 | Reverse: 5- ATTGAAGCATCTGGAGTAATCGTT |
| 1809rt-1 | Forward: 5- CACTTGGCGATGACTGGAGAGG |
| 1809rt-2 | Reverse: 5- CACTTGGCGATGACTGGAGAGG |
| 1776rt-1 | Forward: 5- CCCGAGCGTATTGAGTG |
| 1776rt-2  1819rt-1  1819rt-2  SsePeprt-1  SsePeprt-2  Fhbprt-1  Fhbprt-2  Fhbrt-1  Fhbrt-2  0928rt-1  0928rt-2  0929rt-1  0929rt-2  0930rt-1  0930rt-2  0931rt-1  0931rt-2  0932rt-1  0932rt-2 | Reverse: 5- CAAACCGCAGAGGAAAA  Forward: 5- AGGCTATGCGTGAACTAGGCTACA  Reverse: 5- CTTATCGGACTCGTGCTGGCTATTG  Forward: 5- CTTGTCAACTGGTGGAAAC  Reverse: 5- ATTCTTTGGTGGTGACTTC  Forward: 5- TTCACAACAAAAGAACGCA  Reverse: 5- GCGACAACTTTACCATCAA  Forward: 5- ATAACAGCCTCCTTCCCTA  Reverse: 5- AACGATTGCCTGATTTCC  Forward: 5- ATCCTCTTGATGTTCGCTTAG  Reverse: 5- TTACCCTTTACGGTTGACG  Forward: 5- GTGATTGAGCGTTTGATAGA  Reverse: 5- TTGCGGTTGGCTTAGTC  Forward: 5- GGACAAAGGAGTTCTGGTGGA  Reverse: 5- TTATCGTTAATGGCAAGGTCAA  Forward: 5- TAACCGTATCATAGCGAACC  Reverse: 5- TTACGATAGCGTCACTCCTG  Forward: 5- TGTCGGTTCTTTCTTTGC  Reverse: 5- TGCCAGCGTGCCTTT |
