# Supplementary figures and images for: Streptococcus suis 2 Transcriptional Regulator TstS Stimulates Cytokine Production and Bacteremia to Promote Streptococcal Toxic Shock-Like Syndrome
Source: Front Microbiol. 2018 Jun 19;9:1309. doi: 10.3389/fmicb.2018.01309 (PMC6020791; doi:10.3389/fmicb.2018.01309)

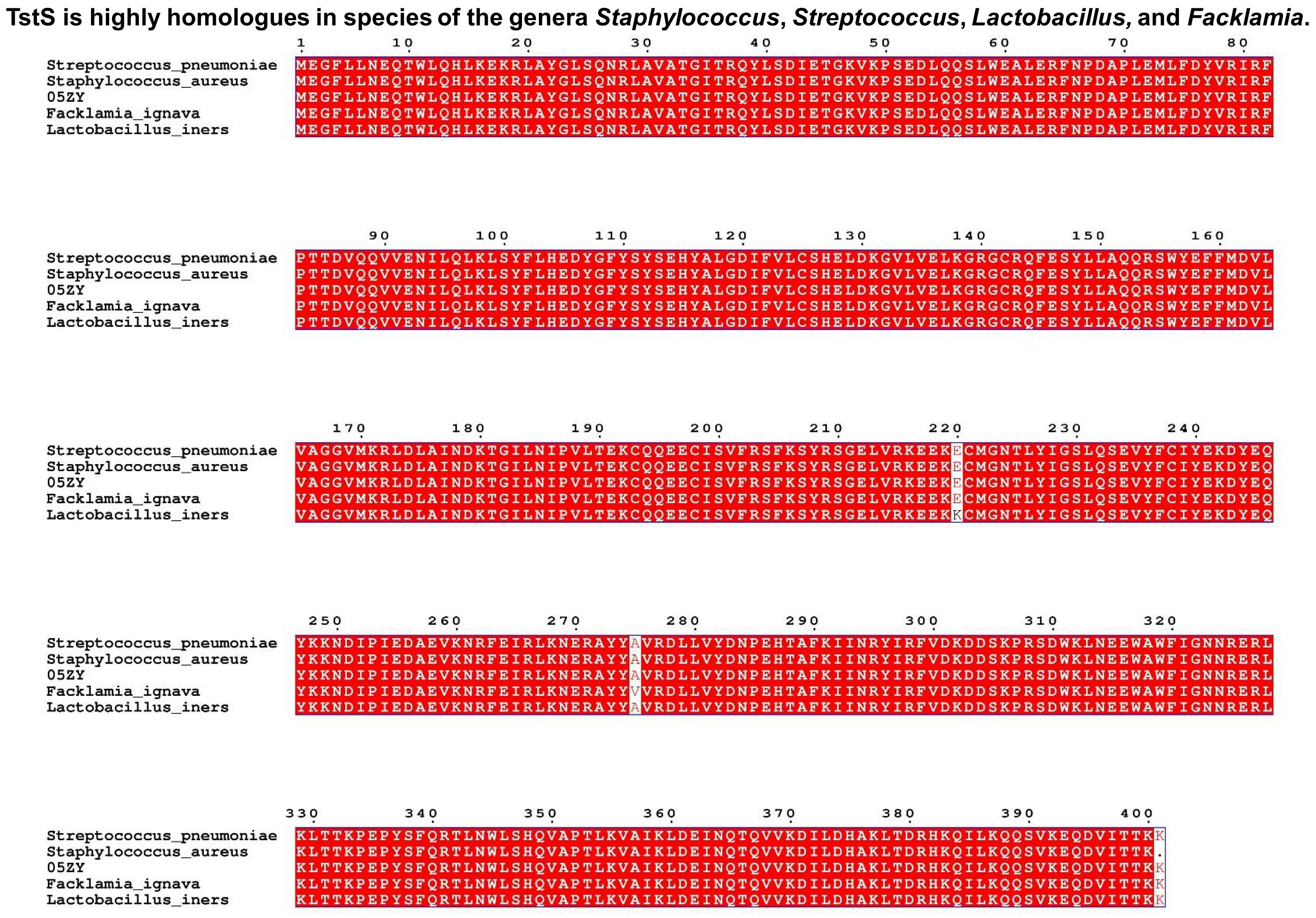

Supplement: Supplementary file 3 [file Image_1.jpg]

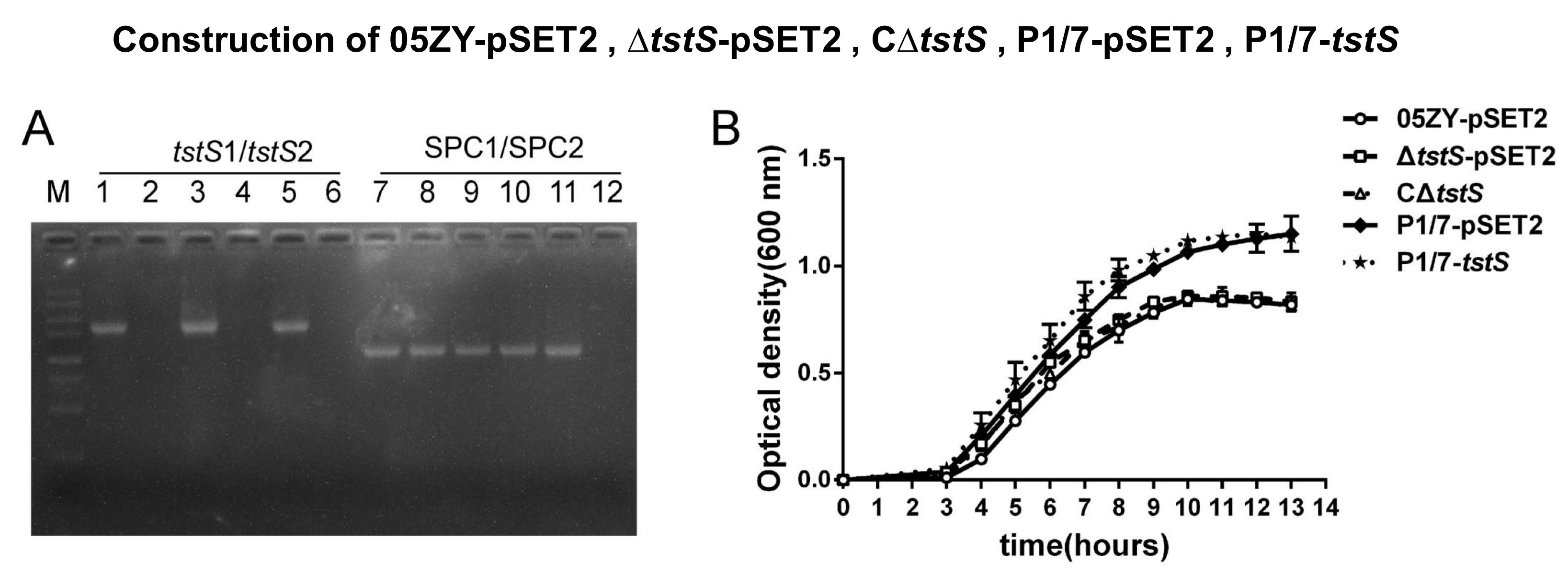

Supplement: Supplementary file 4 [file Image_2.jpg]

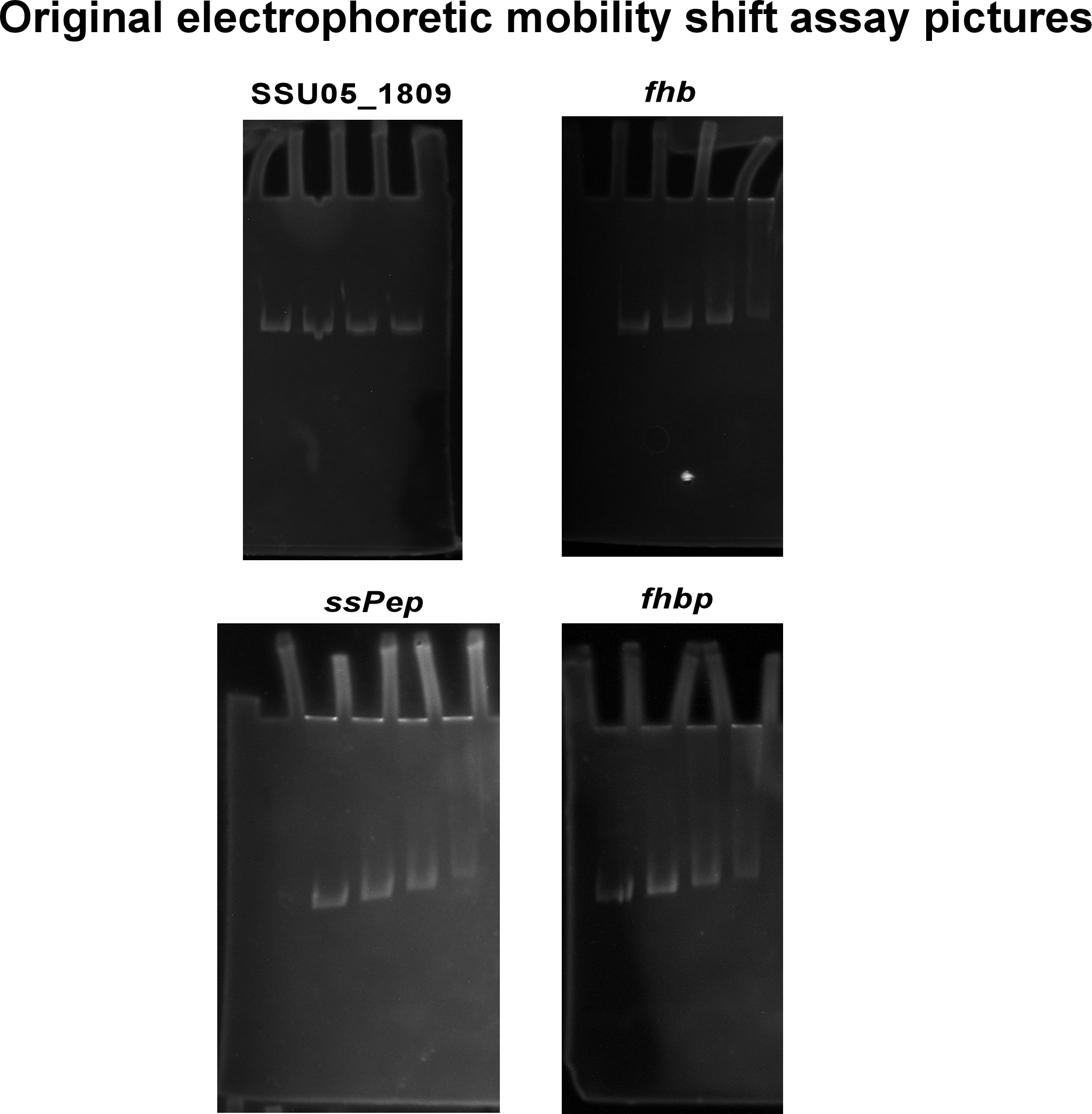

Supplement: Supplementary file 5 [file Image_3.jpg]
